# The Role of Magnetic Resonance Imaging in Preoperative Planning for Patients Undergoing Therapeutic Mammoplasty

**DOI:** 10.1155/2013/260260

**Published:** 2013-12-17

**Authors:** Gareth Hicks, Philip Turton, Sree Rajan, April Nunn, Nisha Sharma, Raj Achuthan

**Affiliations:** Breast Unit, St. James' University Hospital, Beckett Street, Leeds, West Yorkshire LS9 7TF, UK

## Abstract

*Background*. Assessment of the ratio between tumour volume and breast volume in therapeutic mammoplasty is paramount. Traditionally based on clinical assessment and conventional breast imaging, the role of breast magnetic resonance imaging (MRI) in this context has not been established. *Methods*. Data was collected from all women undergoing therapeutic mammoplasty (TM) between 2006 and 2011. Each case was discussed at an MDT where MRI was considered to facilitate surgical planning. The contribution of MRI to disease assessment and surgical outcome was then reviewed. *Results*. 35 women underwent TM, 15 of whom had additional MRI. 33% of patients within the MRI subgroup had abnormalities not seen on either mammography or USS. Of those undergoing MRI, 1/15 patients required completion mastectomy versus 3 patients requiring completion mastectomy and 1 patient requiring further wide local excision (4/20) in the conventional imaging group. No statistical difference was seen between size on MRI and size on mammography versus final histological size, but a general trend for greater correlation between size on MRI and final histological size was seen. *Conclusion*. MRI should be considered in selected patients undergoing therapeutic mammoplasty. Careful planning can identify those who are most likely to benefit from MRI, potentially reducing the need for further surgery.

## 1. Introduction

The primary aim of surgical oncology is complete removal of the cancer with clear margins. Within breast surgery, the realisation that adequate oncological margins could be obtained without full amputation of the breast was first put forward by Keynes in 1937 [1]. With the addition of irradiation of the breast for control of local recurrence in 1939, a fundamental change was brought about in the approach to the breast cancer. With time breast surgery has become less and less radical. The Halstead radical mastectomy and the Patey modified radical mastectomy are now largely confined to the history books and we are without question in an era when the majority of patients can have “breast conserving surgery” [2].

With the refinement of breast conserving surgical techniques, combined with the development of specialist breast surgeon training in reconstructive and aesthetic breast surgery, the place of cosmesis in the surgical management of breast cancer has gained increasing attention. There is evidence that removal of greater than 10–20% of breast volume is associated with unacceptable cosmetic appearance and poor psychological adjustment after surgery [3]. Therefore, the role of oncoplastic surgery in breast cancer has progressively increased in importance since a relative infancy in the 1990s, with an evolution in oncoplastic techniques for the breast, especially the use of reduction mammoplasty approaches to breast cancer management [3]. Although breast reduction in breast cancer management has been in use since the 1980s, it is only since 1998 with its introduction by Audretsch et al. that therapeutic mammoplasty has been clearly defined [4, 5]. Therapeutic mammoplasty (TM) aims to overcome some of the problems associated with breast conserving surgery such as long-term asymmetry, deformity, and technically difficult irradiation of large, ptotic breasts. It may even be of functional benefit to women with macromastia who would otherwise be suitable for reduction mammoplasty [5–7].

Within the preoperative planning of women suitable for TM, the surgeon must consider breast size, tumour location, and tumour size and how the breast will be reconstituted in terms of its shape and the choice of pedicle [6]. In women with an appropriate starting point for TM, it is worthwhile noting that breast conserving surgery can be considered even for tumours larger than 4 cm using this technique.

The role of magnetic resonance imaging (MRI) however in the preoperative diagnosis of breast cancer remains contentious. MRI has been criticised for increasing mastectomy rates, increasing rates of diagnosis of clinically insignificant tumours, and delaying time from diagnosis to treatment [8]. MRI nevertheless has also been shown to have greater sensitivity compared with mammography in high-risk women and in evaluation of the contralateral breast [9, 10]. MRI is accepted therefore to have a role in the assessment of high-risk women, the characterisation of uncertain lesions and in the evaluation of residual disease after lumpectomy [11, 12]. Current recommendations support its use in selected roles but the contribution of MRI to the preoperative planning of therapeutic mammoplasty remains undefined.

## 2. Objectives

To assess the role and contribution of MRI to the preoperative planning of women with breast cancer who are considered potentially suitable for therapeutic mammoplasty.

## 3. Methods

All women who underwent TM between 2006 and 2011 were identified from a prospectively collected database at our institution. Patients suitable for TM were assessed by the reconstructive breast surgeon at the Leeds Breast MDT where on an individual case by case basis, the decision for preoperative MRI was made.

Data was reviewed regarding patient age, tumour type, palpability, and findings on mammography and sonography. Further data regarding multifocality, final histological size and weight, use of neoadjuvant chemotherapy, need for further surgery, tumour size as determined by MRI, and any supplementary MRI findings compared with mammography or sonography was collected. Finally, the clinical utility of MRI as regards preoperative planning was recorded and the impact MRI had on the choice of index operation and potential need for further surgery also.

## 4. Results

Statistics were calculated using GraphPad Prism 5 (GraphPad Software, La Jolla, CA, USA). Data was treated as nonparametric and recorded as median and interquartile range (IQR). Comparison between groups has been carried out with the Mann-Whitney *U*-test for categorical data, with the chi-squared test comparing observed and expected frequencies. Spearman's test has been used for correlation.

Thirty-five women underwent TM of whom 15 (43%) had preoperative MRI in addition to conventional imaging. Baseline patient and tumour characteristics are shown in Table 1.

preoperative tumour size as measured by mammogram, USS, and MRI was generally greater in the conventional imaging only group, as was final histological size and specimen mass (Table 2).

Of the 15 women who underwent preoperative MRI, five were noted to have an additional area of malignant enhancement not seen on conventional imaging. The management of four of these patients was altered to take account of the previously unrecognised lesions. The information was specifically used by the surgeon as part of the surgical planning for the TM to incorporate additional volume resection and enable breast reshaping (Table 3).

Results of chi-squared analysis showed that there was no statistical difference in the numbers of palpable tumours (*P* = 0.67), multifocal tumours (*P* = 0.67), or those whose tumours contained DCIS (*P* = 0.72) between the two groups (Table 4). Mann-Whitney *U* analysis did not show a significant difference between the median specimen mass of the conventional imaging + MRI group and the conventional imaging alone group (*P* = 0.84).

Overall 14% patients (5/35) required further surgery after TM. In the group who had conventional imaging and MRI, just one patient (7%, 1/15) required further surgery. By contrast in the conventional imaging group four (20%, 4/20), patients required further surgery although this was not statistically significant (chi-squared test: *P* = 0.53); figures for relative risk and odds ratio were 0.33 and 0.36, respectively.

There was no significant difference in final histological size between the resected specimens of patients who had undergone MRI planning and those who have had conventional imaging (chi squared); however, there was better correlation between the size on MRI and the final histologically reported invasive tumour size. The degree of correlation appeared less robust when comparing MRI size and the whole tumour size when DCIS was taken into consideration. (Table 5).

At one year, no patient had had recurrence of their disease. To date (October 2012), five patients have had recurrence of their disease and one patient has been lost to followup. Of those with recurrence, one patient had liver metastases at 29 months, one patient had bony metastases at 38 months, one patient had axillary recurrence at 38 months, and one patient had a brain metastasis at 21 months who subsequently died at 30 months after surgery. One patient had a local recurrence at 28 months (despite clear margins in shavings). Disease-free survival was 80% in the conventional imaging plus MRI group and 90% in the conventional imaging alone group (*P* = 0.81, chi squared).

## 5. Discussion

The role of MRI in the assessment of patients with breast cancer continues to defined. Whilst MRI has been shown to be give a better estimation of intraductal spread and therefore estimation of size of disease, it may also overestimate tumour size, or misclassify benign lesions as being malignant [13–17]. The COMICE trial gave evidence that the addition of MRI to preoperative imaging does not reduce reoperation rates [18]. The COMICE study, however, had the drawback of lack of experience with preoperative breast MRI, especially by surgeons and in the use of MR-guided procedures. Additionally, the study design was based on a cohort already selected for breast conservation therapy [18]. In an era when ever larger lesions are beginning to be considered suitable for breast conserving surgery, the additional information gleaned by preoperative MRI may allow women with borderline lesions (with respect to size) to be considered, thus reducing the risk of unnecessary mastectomy. A better estimation of the size of a lesion may also reduce the likelihood of larger than necessary volume resections during TM, where cosmetic appearance is a key facet of the operation. Our study was therefore important to verify the practical utility of MRI in the setting of consideration for TM.

Our results have shown that MRI correlates more closely with the final histological size than mammography in the assessment of invasive carcinoma. Whilst this may be useful in reducing the volume of excision, MRI was less accurate in its ability to predict whole tumour volume in relation to DCIS. There is some limited evidence that MRI may be better than mammography alone in predicting size of DCIS; however, no modality is yet capable of giving acceptable sensitivity and specificity as to the size of DCIS [19].

Of the 15 patients who underwent MRI, 30% were found to have additional lesions (5/15) in whom additional cancer was identified in 60% (3/5). Management of these patients was altered to take account of these lesions (enlarging the extent of the wide local excision, converting from a proposed wide local excision to quadrantectomy, or extending the resection into adjacent quadrants to ensure negative margins). Margin positivity resulted in further surgery in 7% (1 patient) of the conventional imaging plus MRI group compared to 20% (4 patients) in the conventional imaging alone group. Relative risk and odds ratio figures of 0.33 and 0.36 would infer a potential risk reduction with preoperative MRI; however, given the small numbers in our study, this may simply represent a type 1 error. Conversely, the lack of statistical significance may imply a type 2 error, again reflecting the need for greater patient numbers. Of importance, we have shown that the mass, and therefore by implication the volume of resected tissue, did not significantly increase in the conventional imaging plus MRI group, suggesting that MRI is guiding surgical planning, providing a useful road map of the areas of concern to be excised rather than bluntly suggesting that a larger volume excision is required.

Our reoperation rates were somewhat higher than other published data for therapeutic mammoplasty specifically (14% versus 2%) but lower than the rates for all forms of breast conserving surgery (20% versus 14%) [20, 21]. This improvement in accuracy of surgical excision has likely aided our low repeat surgery rates in the MRI group.

Our rates of margin positivity and local recurrence are in line with recent published literature (McIntosh and O'Donoghue, Meretoja et al.) [20, 21]. The completed follow-up period for all patients was relatively short at twelve months, although we note that the use of MRI and therapeutic mammoplasty had no negative impact on surveillance mammography.

The cohort of patients who underwent preoperative MRI planning in our study had a smaller volume of tissue resected than those who had conventional image planning. Whilst some centres have reported excellent rates of margin positivity with conventional imaging, others have reported markedly higher rates [20, 22]. It stands to reason that increased volume of excision reduces the likelihood of a positive margin, but volume excised must be balanced against volume required for reconstruction in TM. MRI would therefore seem to show promise to provide the operating surgeon with more information to safely reduce the volume of excised tissue. We propose to evaluate this hypothesis with further data collection and a more prolonged period of followup.

Thus, the role of MRI maybe surmised as a useful tool to aid in decreasing the number of women requiring further surgery following therapeutic mammoplasty. It is particularly helpful where the extent of a tumour is difficult to determine such as in cases of lobular breast carcinoma and where additional lesions are found requiring further characterisation.

The selective use of MRI can therefore aid the multidisciplinary team in assessing suitability for therapeutic mammoplasty, help avoid excessive surgery, achieve negative resection margins, and reduce requirement for reoperation for margin positivity.

## 6. Conclusion

MRI should be used selectively in addition to conventional breast imaging for surgical planning in patients considered for therapeutic mammoplasty. It can assist in reducing unnecessarily large volume resections, help achieve negative margins, and reduce reoperation rates.

## Figures and Tables

**Table 1:** 

	Conventional imaging + MRI	Conventional imaging only
Mean age (years) (range)	52.2 (43–62)	56.7 (37–77)

Multifocal tumour	4	3

Neoadjuvant chemotherapy	4	0

Tumour type (assessed on final histology)	Invasive ductal: 3	Invasive ductal: 3
	Invasive and DCIS: 6	Invasive and DCIS: 11
	DCIS alone: 3	DCIS alone: 3
	Lobular: 1	Lobular: 1
	Complete pathological response to NACT: 2	Phyllodes: 2

**Table 2:** 

	Conventional imaging + MRI (interquartile range)	Conventional imaging only (interquartile range)
Median size on MRI (mm)	26 (20)	
Median size on mammography (mm)	12 (28.5)	23.5 (19.75)
Median size on ultrasound (mm)	12 (21.5)	20 (15.25)
Median size on final histology (mm)	23 (18.5)	25.5 (21)
Median specimen mass (g)	187 (178)	213 (493)
Further surgery	1 patient required completion mastectomy for margin positivity	3 patients required completion mastectomy 1 patient required further wide local excision for margin positivity

**Table 3:** 

	MRI findings	Findings on further investigation	Effect on surgical management
1	Focal 10 mm tumour on conventional imaging but mixed intraductal/lobular; MRI was performed where 2 satellite lesions were noted	2nd look USS—one C4 lesion, other B2 but not diagnostic	3 tumours excised all within primary specimen (10, 2, and 1 mm)

2	MRI identified additional lesion not seen initially	2nd look USS identified second lesion, diagnostic biopsy—B1. On excision—B5b	Main 14 mm tumour mass with separate 1.2 mm satellite lesion (whole tumour 21 mm)

3	MRI showed initial 9 mm tumour with further suspicious nodule 3 cm away	2nd nodule not seen on second look USS. Diagnostic biopsy—B5b	Nodule included in resection specimen allowing successful single-stage surgery

4	MRI confirmed 12 mm mass and suggested second lesion	2nd look USS could not clearly identify lesion but fine needle aspirate—C3 (fibroadenoma with atypia)	13 mm invasive tumour with second nodule confirmed benign lesion (fibroadenoma)

5	MRI suggested extent of disease greater than that suggested by conventional imaging. It was also suggestive of a lesion in the contralateral breast	Second look USS—normal	No impact

**Table 4:** 

	Palpability		Multifocality		DCIS	
	Palpable	Impalpable	Multifocal tumour	Single tumour	DCIS	No DCIS
Conventional imaging + MRI	7	8	4	11	8	7
Conventional imaging only	12	8	3	17	15	5

**Table 5:** Correlation between invasive and whole tumour size versus size on final histology^∗, ∗∗^.

MRI		Conventional imaging	
Invasive tumour	Whole tumour	Invasive tumour	Whole tumour
0.61	−0.14	0.29	0.1

*Final histological size for those who had neoadjuvant chemotherapy was taken as size on MRI.

**Spearman's rank coefficient.
